# Role of Non-Coding RNAs in TGF-β Signalling in Glioma

**DOI:** 10.3390/brainsci13101376

**Published:** 2023-09-27

**Authors:** Bakhya Shree, Vivek Sharma

**Affiliations:** Department of Biological Sciences, Birla Institute of Technology and Science, Pilani-Hyderabad Campus, Jawahar Nagar, Hyderabad 500078, India; bakhyashree@gmail.com

**Keywords:** lncRNA, miRNA, circRNA, GBM, TGF-β

## Abstract

Brain tumours and Gliomas, in particular, are among the primary causes of cancer mortality worldwide. Glioma diagnosis and therapy have not significantly improved despite decades of efforts. Autocrine TGF-β signalling promotes glioma proliferation, invasion, epithelial-to-mesenchymal transition (EMT), and drug resistance. Non-coding RNAs such as miRNA, lncRNA, and circRNAs have emerged as critical transcriptional and post-transcriptional regulators of TGF-β pathway components in glioma. Here, we summarize the complex regulatory network among regulatory ncRNAs and TGF-β pathway during Glioma pathogenesis and discuss their role as potential therapeutic targets for Gliomas.

## 1. Introduction

### 1.1. Gliomas

Gliomas are a group of brain tumours clinically divided into four types from grade I to grade IV. Grade IV gliomas, known as Glioblastoma multiforme (GBM), are the most common form of adult brain cancer [1,2]. The etiology of GBMs is complex and involves mutation or overexpression of multiple genes, and they have high intra-tumour heterogeneity [3,4]. Based on the molecular characteristics of GBM, the World Health Organization (WHO) classifies it into three types: GBM isocitrate dehydrogenase (*IDH*) wild type, GBM *IDH* mutant, and GBM not otherwise specified (NOS) [5,6]. Mutations in the *IDH* group of genes represent the most critical genetic alterations in GBM, which plays a vital role in therapeutic responses [7]. GBMs likely originate from astrocytes; however, tracking the cell of origin in GBM is challenging due to their heterogeneity [8]. Diffuse intrinsic pontine glioma (DIPG) is a form of pediatric malignancy that primarily grows in the pons with a dismal prognosis [9]. DIPG shares resemblances with adult high-grade astrocytomas. However, this has been debated lately due to its distinct molecular alterations [9,10]. Glioma stem cells (GSCs) are glioma-initiating cells that form a small subpopulation of GBM tumour cells and express stemness markers, such as *CD133* [8]. GSCs can differentiate into multiple tumour cell types, contributing to intratumour heterogeneity in GBM [4]. GSCs contribute to tumour initiation, therapeutic resistance, and recurrence [8].

Current therapeutic strategies for GBM include maximum surgical resection and radio- and chemotherapy with temozolomide (TMZ) [11,12]. TMZ is an oral alkylating agent that alkylates DNA bases; it causes mismatch during DNA replication, leading to futile rounds of DNA repair, DNA double-strand breaks, and apoptosis [13]. However, O6-methylguanine-DNA methyltransferase (*MGMT*) can resolve some TMZ-induced alterations and thus mediate the survival of GBM cells [14]. *MGMT* inhibitors are considered beneficial for improving the action of TMZ in GBM patients [15]. Localized application of pseudo-substrates or tumour-specific delivery of blocking peptides against *MGMT* increases TMZ efficiency [14]. Overall, TMZ treatment extends the survival of GBM patients from 12.1 to 14.6 months [11], while tumour recurrence in GBM patients is inevitable. Resistance to radiation and chemotherapy in gliomas is also due to various other adaptive mechanisms, such as enhanced DNA repair capacity, cytoprotective autophagy, deregulated signalling pathways, intratumoral heterogeneity, phenotypic plasticity, and hypoxia [16].

### 1.2. Transforming Growth Factor-β (TGF-β)

TGF-β is a pleiotropic cytokine that regulates cell proliferation, differentiation, tissue homeostasis, motility, invasion, extracellular matrix production, angiogenesis, epithelial to mesenchyme transition (EMT), chemoresistance, and immune response in various cancers, including GBM [17,18]. TGF-β also contributes to pathologies associated with virus and bacterial infections as an inflammatory cytokine [19]. TGF-β superfamily consists of a large spectrum of ligands, including TGF-β1, TGF-β2, TGF-β3, activin, nodal, bone morphogenetic protein (BMP), growth and differentiation factors (GDF), and anti-mullerian hormone (AMH) [20]. Seven types I and five types II transmembrane serine/threonine-protein kinase receptors exist for the TGF-β superfamily in the mammalian genome [21]. TGF-β ligand-mediated signalling is initiated by the binding of TGF-β to the type II TGF-β receptor (TGF-βR II), which gets phosphorylated, alters its conformation, and phosphorylates the type I TGF-β receptor (TGF-βR1). The signal through TGFβR1 is transduced downstream either through SMAD proteins—in the canonical TGF-β pathway or through other effectors like *MAPK*, *ERK*, and *JUN kinase*—in the non-canonical TGF-β pathway [20]. TGF-β target genes consist of evolutionarily conserved putative SMAD binding elements (SBEs) in their promoter regions. Nuclear translocation of the activated SMAD2/3 complex and binding of the translocated SMAD2/3 complex to the SBEs leads to the activation or repression of hundreds of *TGF-β* target genes [22]. Non-canonical or non-*SMAD* pathways include various branches of *MAP* kinase pathways, Rho-like GTPase signalling pathways, and phosphatidylinositol-3-kinase/*AKT* pathways. For example, in the *ERK*-mediated signal transduction, the activated TGFβRI phosphorylates and activates the ShcA protein, forming the *SHCA/GRB22/SOS* complex, followed by the sequential activation of *c-RAF*, *MEK*, *ERK* [23]. In normal cells and the early stages of cancer, TGF-β restrains cell proliferation whereas, in advanced stages of cancer, due to accumulation of mutations in the TGF-β pathway components or selective impairment of its tumour-suppressive function, it turns out to be oncogenic [24,25,26]. TGF-β levels are elevated in glioma and are associated with increased histologic grade [27,28]. TGF-β expression is also higher in the serum of GBM patients and correlates with poor survival [29]. TGF-β promotes TMZ resistance by activating genes, such as connective tissue growth factor (*CTGF*), *ZEB1*, and *SNAIL1* [30,31,32]. TGF-β also promotes TMZ resistance in MGMT promoter hypomethylated GBM [33]. Several anti-TGF-β antibodies, inhibitors, and antisense oligonucleotides (ASOs) against TGF-β pathway components have been evaluated in the pre-clinical and clinical trials for GBM, with limited success [18,34,35,36]. Systemic inhibition of TGF-β may not be ideal as it has both pro and anti-tumour activities and might also hamper the normal physiological functions of the TGF-β pathway.

### 1.3. Non-Coding RNAs (ncRNAs)

The human genome may be categorized into protein-coding genes (PCGs) and non-protein-coding genes (NCGs). ncRNAs constitute >90% of the human genome. The non-coding category of the genome is highly heterogeneous, consisting of small non-coding RNAs < 200 bps in length and long ncRNAs (lncRNAs) > 200 bps in length. 

#### 1.3.1. miRNAs

miRNAs are small non-coding RNAs of 21–23 nucleotides in length that generally perform post-transcriptional gene silencing of their mRNA targets [37]. They regulate gene expression by primarily interacting with the miRNA response elements (MREs) present on the 3′ UTR of a transcript. However, interaction with 5′ UTR, coding sequences, and gene promoters are also observed [38]. They bind to the target gene and either degrade the transcript or, more often, limit the target gene’s translation [39]. The mRNA decay by miRNA occurs through the miRNA-induced silencing complex (miRISC) [40]. Argonaute and GW182 are the core components of the RISC complex. GW182 interacts with PABP and recruits the PAN2-PAN3 and CCR4-NOT deadenylase complex, causing deadenylation, decapping, and mRNA decay [41]. miRNAs also prevent protein synthesis by cap-dependent translational inhibition [42]. It interferes with the assembly or activity of the translation initiation complex, eIF4F, via eIF4E-T and DDX6 [42]. While these are the canonical miRISC-mediated silencing mechanisms, there are few non-canonical miRNA-mediated gene silencing. For example, complete sequence complementarity between miRNA and target mRNA results in direct AGO2-mediated target cleavage [43]. Also, the recruitment of Argonaute in the absence of GW182 inhibits translation without affecting mRNA stability [44]. The differential association of Argonaute protein with other proteins mainly decides the outcome of mRNA decay or translation inhibition [40]. Several miRNAs regulate multiple aspects of GBM pathogenesis and are potential diagnostic biomarkers and therapeutic targets for GBM [45]. 

#### 1.3.2. LncRNAs

LncRNAs are transcripts longer than 200 bps with no ability to code for proteins [46,47,48,49,50,51,52,53]. More than 100,000 lncRNAs in the human genome are listed in the NONCODE and other lncRNA databases [50,54]. While most lncRNAs are generated from RNA polymerase II, which shares similarities with mRNAs, such as polyadenylation and 7-methylguanosine cap, some are generated from RNA pol I and RNA polymerase III [50]. LncRNAs have a wide range of functions to modulate gene expression, chromatin architecture, transcription, RNA processing, splicing, editing, localization, stability, and protein translation [50]. They modulate gene expression in *cis* and *trans* by interacting with DNA, RNAs, and proteins [55]. To regulate gene expression, lncRNAs modulate (i) recruitment of a regulatory/transcription factor/epigenetic protein to a gene locus; (ii) inhibit the binding of a transcription factor to gene promoter by acting as a decoy; (iii) by acting as scaffold for protein complexes to either positively or negatively regulate gene expression; and (iv) large number of lncRNAs localized in the cytoplasm function as competing endogenous RNAs (ceRNAs) for miRNAs and stabilize the mRNA target of those miRNAs. LncRNAs regulate multiple aspects of GBM pathogenesis, such as proliferation, invasion, metastasis, and drug resistance [56]. They have the potential to serve as potential diagnostic markers and therapeutic targets for GBM [57,58]. 

#### 1.3.3. CircRNAs

Circular RNAs are covalently closed, single-stranded RNAs produced by a non-canonical back splicing of cellular non-coding RNAs and precursor messenger RNAs [54,55,56]. CircRNAs are generated in the nucleus, but most are primarily present in the cytoplasm. Their synthesis is regulated by specific *cis*-acting elements and *trans*-acting factors [59,60,61,62,63]. Many circRNAs act as non-coding RNAs and regulate gene expression by serving as decoys or competitors for microRNAs and proteins. In addition to sponging miRNAs and proteins, circRNAs also regulate the splicing of linear RNAs, regulate transcription, and form chromatin looping [60,64] A small percentage of circRNAs undergo cap-independent translation to encode functional peptides in response to specific cellular stresses [61]. CircRNAs regulate proliferation, angiogenesis, cancer cell migration and invasion, and apoptosis in cancer [62]. CircRNAs may act as diagnostic biomarkers and therapeutic targets in several cancer types, including GBM [62,63,65,66].

The miRNAs, lncRNAs, and circRNAs are aberrantly expressed in GBM tumour tissues and regulate GBM pathogenesis (Table 1, Table 2 and Table 3) [57,65,67]. They play critical regulatory functions in cancer by acting as oncogenes or tumour suppressors [68,69,70,71]. They have the potential for use as clinical biomarkers and therapeutic targets for cancers, including GBM [72,73,74,75,76,77].

**Figure 1:**
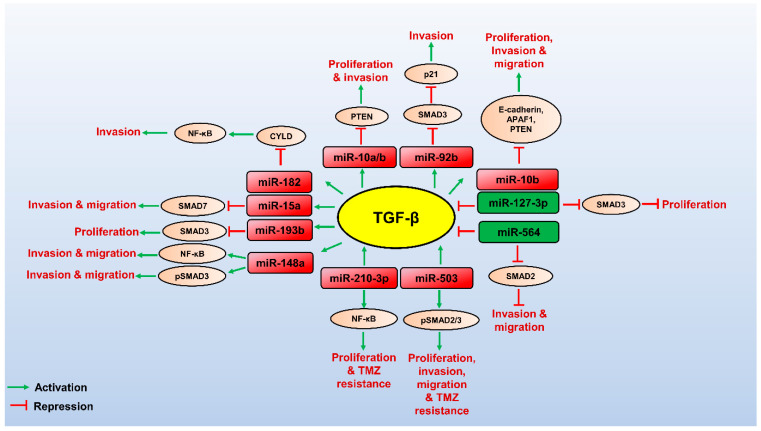
Role miRNAs and their targets in regulating the TGF-β pathway in GBM. TGF-β (yellow circled) promotes the expression of oncogenic miRNAs (red boxed), which can control post-transcriptional gene expression of its targets (brown circled) to promote TGF-β-mediated GBM pathogenesis. Few tumour suppressor miRNAs (green boxed) target the TGF-β pathway’s components and downregulate the TGF-β signalling, thereby attenuating GBM progression. The red arrows indicate inhibitory function, and the green arrows indicate a stimulatory role.

**Figure 2:**
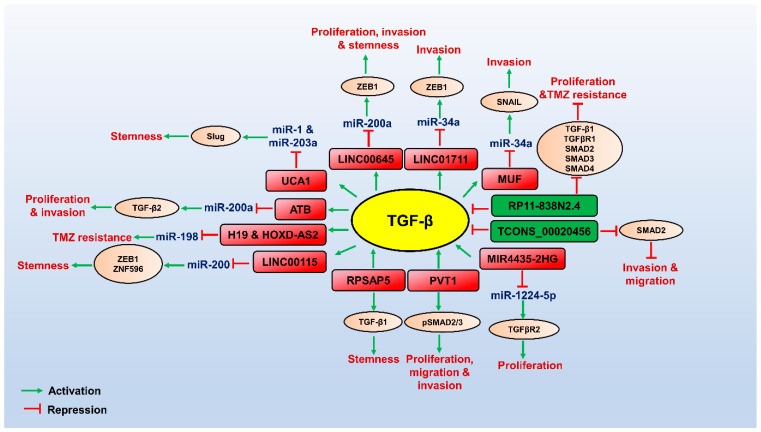
Role of LncRNAs involved in TGF-β pathway in regulating GBM pathogenesis. TGF-β (yellow circled) promotes the expression of oncogenic lncRNAs (red boxed), which can control transcriptional/post-transcriptional gene expression of its targets (brown circled) by interacting with miRNAs or proteins to promote TGF-β-mediated GBM pathogenesis. Few oncogenic lncRNAs are not induced by TGF-β but promote the TGF-β signalling-mediated GBM pathogenesis. Tumour suppressor lncRNAs (green boxed) target the components of the TGF-β pathway and downregulate the TGF-β signalling, thereby attenuating GBM progression. The red arrows indicate inhibitory function, and the green arrows indicate a stimulatory role.

**Figure 3:**
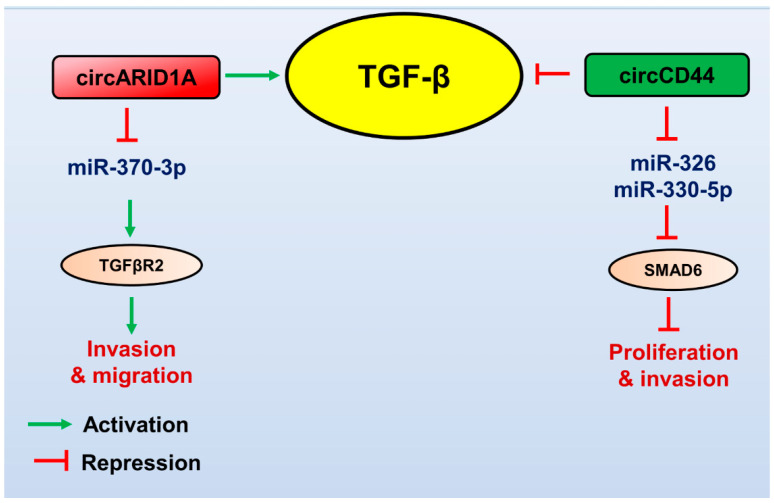
Role of circular RNAs in regulating the TGF-β pathway in GBM. TGF-β (yellow circled) promotes the expression of oncogenic circRNA (red boxed), which controls the gene expression of its targets (brown circled) to promote TGF-β-mediated GBM pathogenesis. Tumour suppressor circRNA (green boxed) targets the TGF-β pathway’s components, downregulates the TGF-β signalling, and attenuates GBM progression. The red arrows indicate inhibitory function, and the green arrows indicate a stimulatory role.

Several miRNAs, lncRNAs, and circRNAs have recently been identified, which modulate the TGF-β pathway to promote or repress GBM (Table 1, Table 2 and Table 3). Since TGF-β plays a role in tumour suppression at early stages of cancer development, its complete inactivation for cancer treatment is not ideal [24,25,26]. NcRNAs regulated by the TGF-β pathway modulate numerous aspects of GBM pathogenesis. They may serve as attractive therapeutic targets downstream of TGF-β for countering the tumour-promoting effects of TGF-β. Here, we summarize the role of lncRNAs, miRNAs, and circRNAs in the TGF-β pathway in GBM pathogenesis (Figure 1, Figure 2 and Figure 3).

## 2. miRNAs Involved in the TGF-β Pathway in GBM 

### 2.1. Oncogenic miRNAs Involved in the TGF-β Pathway in Gliomas

#### 2.1.1. *miR-182*

*miR-182* is over-expressed in GBM tissues and cells, whereas CYLD, a negative regulator of the NFκB pathway, is downregulated [78]. The genomic location of *miR-182*, 7q32.1, is frequently amplified in gliomas [78]. Interestingly, *miR-182* is induced by TGF-β in U373MG and LN229 cells through the SMAD signalling pathway [78]. *miR-182* expression was also upregulated in Smad2/Smad4- overexpressing cells [78]. ChIP assays confirmed the binding of SMAD2/3 to the promoter of *miR-182* [78]. These results suggest that TGF-β induced *miR-182* expression in glioma cells through the SMAD signalling pathway. CYLD is a target of *miR-182* [78]. Up-regulation of *miR-182* in U373MG and LN229 cells decreased the expression of CYLD. Also, *miR-182* interacts with and degrades CYLD. Further, overexpression of *miR-182* increased, while inhibition of *miR-182* reduced the luciferase activity of NF-κB reporter and expression of NF-κB target genes. *miR-182* overexpression significantly induced the phosphorylation of IKKβ, while *miR-182* inhibitor reversed this effect. Importantly, in vitro kinase assay showed that endogenous IKK kinase activity was prolonged in *miR-182* –transduced cells. These results indicate that *miR-182* suppresses CYLD and enhances and sustains NF-κB activity in GBM [78]. *miR-182* up-regulation promotes anchorage-independent growth, colony formation, and invasion of GBM in vitro and in vivo [78]. At the same time, *miR-182* inhibition reversed these effects [78]. Also, the tumour-promoting function of *miR-182* overexpression was reversed with combined transfection of *miR-182* mimics and IκBα dominant-negative mutant construct, indicating that *miR-182* promotes GBM tumour through the NF-κB pathway [78]. Further, *miR-182* suppression inhibits NF-κB activity and malignant properties of patient-derived glioma cells (PDGCs) [78]. The study also identified that TGF-β treatment in U373MG and LN229 cells significantly increased the NF-κB reporter activity, which was abolished upon *miR-182* inhibition. This indicates that TGF-β-induced *miR-182* is essential for sustained NF-κB activity in GBM [78]. Overexpression of pSMAD2/3, *miR-182*, and several NF-κB target genes was observed in clinical GBM samples, which conferred poor survival of the patients [78]. Also, the clinical samples showed a positive correlation between TGF-β, *miR-182*, and NF-κB target genes [78]. Upon induction by TGF-β, *miR-182* promotes GBM pathogenesis by activating and sustaining NF-κB activity by downregulating CYLD [78]. 

#### 2.1.2. *miR-15a*

Guo et al., using a microarray screen, observed that *miR-15a-5p* is upregulated in glioma tissues [79]. Bioinformatics analysis and luciferase reporter assay confirmed the direct interaction of *miR-15a* with SMAD7. Overexpression of *miR-15a* promoted migration and invasion of SHG139 cells [79]. Whereas inhibition of *miR-15a* displayed the opposite effect, indicating the tumour-promoting ability of *miR-15a* [79]. Anti-miRNA oligonucleotide (AMO)-mediated inhibition of *miR-15a* in SHG139 cells reduced migration and downregulated mesenchymal markers—Vimentin and N-cadherin. However, the combined knockdown of *miR-15a* and SMAD7 reversed these effects [79]. Further, *miR-15a* inhibition attenuated GBM tumour growth in vivo. Hence, this study indicates the oncogenic function of *miR-15a* in GBM by inhibiting SMAD7 activity [79]. 

#### 2.1.3. *miR-193b*

*miR-193b* levels are upregulated in glioma cell lines and GBM tumour samples [80]. *miR-193b* depletion reduces the proliferation of U87 and U251 cells [80]. Cell cycle analysis upon *miR-193b* knockdown revealed G0/G1 arrest in U87 and U251 cells [80]. Bioinformatics analysis and luciferase reporter assays demonstrated that SMAD3 is the primary target of *miR-193b* [80]. Inhibition of *miR-193b* significantly increased the protein levels of SMAD3 in U87 and U251 cells [80]. Further, to know whether *miR-193b* regulates GBM proliferation through the TGF-β pathway, one of the primary targets of the TGF-β pathway, p21 levels were evaluated upon *miR-193b* inhibition. Inhibition of *miR-193b* and the subsequent up-regulation of SMAD3 in GBM cells displayed a significant accumulation of p21 [80]. Also, the down-regulation of miR-193b decreased the proliferation of U87 and U251 cells [80]. Hence, the study demonstrates that *miR-193b* is an oncogene that promotes cell growth by directly targeting SMAD3 and restricting the tumour-suppressive effects of SMAD3 through p21 down-regulation in GBM [80]. 

#### 2.1.4. *miR-210-3p*

*miR-210-3p* is induced upon exposure to hypoxic conditions in GBM [81]. Treatment of the hypoxia-exposed cells with echinomycin, a transcriptional inhibitor of *HIF-1α*, significantly reduced hypoxia-induced *miR-210* levels, indicating the transcriptional activation of *miR-210-3p* by HIF-1α in GBM [81]. *HIF-1α* promotes TGF-β expression, which was abrogated upon *miR-210-3p* inhibition. This indicates that *miR-210-3p* is an essential mediator in promoting HIF-1α-mediated TGF-β expression [81]. Exposure of U87-MG cells to hypoxic conditions and TGF-β individually promoted migration and invasion. However, this effect was abrogated upon *miR-210-3p* inhibition, indicating an oncogenic role of *miR-210-3p* in promoting hypoxia/TGF-β -mediated GBM pathogenesis [81]. Overexpression of *miR-210-3p* promotes resistance to TMZ in U87-MG cells. However, the chemoresistance induced by *miR-210-3p* overexpression was abrogated upon TGF-β knockdown [81]. Hypoxia-induced *miR-210-3p* also promotes the transcription activity of NF-κB in U87-MG cells [81]. This study establishes that HIF-1α-induced *miR-210-3p* promotes the TGF-β expression and TGF-β-mediated migration, invasion, and TMZ resistance in GBM [81]. However, further studies on the role of *miR-210-3p* on NF-κB-mediated GBM pathogenesis and the involvement of TGF-β in this context are yet to be studied. 

#### 2.1.5. *miR-148a*

Quaking (*QKI*), an essential negative regulator of the TGF-β signalling, is downregulated, and *miR-148a* is upregulated in GBM tissues and cell lines [82]. Low levels of *QKI* and overexpression of *miR-148a* confer poor prognosis in GBM patients [82]. Microribonucleoprotein (miRNP) IP and luciferase reporter assay confirmed the interaction between *miR-148a* and *QKI* [82]. Overexpression of *miR-148a* reduced the protein levels of QKI, while inhibition of *miR-148a* reduced this effect [82]. Moreover, *SKP1*, a member of the E3 ubiquitin ligase complex that degrades SMAD3, is also an essential target of *miR-148a* [82]. Consequently, *miR-148a* overexpression enhanced the SMAD luciferase reporter activity and phosphorylation of SMAD2/3 [82]. GSEA and TCGA database analysis revealed that *miR-148a* positively correlates with the gene signatures of TGF-β signalling [82]. Overexpression of *miR-148a* is associated with poor prognosis in GBM patients, indicating that *miR-148a* is an oncogenic miRNA [82]. High levels of *miR-148a* are positively correlated with p-SMAD3 expression and the DNA binding activity of NF-κB [82]. Also, the promoter of *miR-148a* contains multiple NF-κB binding domains. Consequently, NF-κB activation increased the expression of *miR-148a* in GBM cells [82]. Further, in vitro experiments in LN18 and U-138MG cells and in vivo experiments demonstrated that *miR-148a* promotes the invasion, migration, and aggressiveness of GBM [82]. These results illustrate that the hyperactive NF-κB signalling in GBM promotes the expression of *miR-148a* oncogene, which further promotes the aggressive phenotypes of GBM by downregulating the expression of QKI and SKP1 and activating the TGF-β signalling [82]. Thus, *miR-148a* establishes an essential link between NF-κB and TGF-β signalling in promoting GBM pathogenesis [82]. 

#### 2.1.6. *miR-10a/b*

Liu et al. observed a significant positive correlation between the expression of *miR-10a/b* and TGF-β in glioma tissues [83]. *miR-10a/10b* is induced upon TGF-β treatment of U251 and SHG-44 cells [83]. *miR-10a/10b* overexpression significantly increased the migration of U251 cells [83]. Bioinformatics analysis and luciferase reporter assay revealed that *PTEN*, an important tumour suppressor gene, is a primary target of *miR-10a/10b* [83]. Also, overexpression of *miR-10a/10b* reduced the expression of *PTEN*, while depletion of *miR-10a/10b* displayed the reverse effect. These results indicate that *miR-10a/10b*, upon induction by TGF-β, promotes GBM pathogenesis by inhibiting *PTEN* [83]. 

#### 2.1.7. *miR-10b*

*miR-10b* expression is upregulated upon TGF-β treatment in U87 and U251 cells through the SMAD signalling pathway [84]. Also, the induction of *miR-10b* upon TGF-β treatment in U251 cells was abrogated considerably upon treatment with TGF-β inhibitor SB431452 [84]. *miR-10b* mimics increased the proliferation in GBM [84]. The combination of anti-*miR-10b* and TGF-β treatment reversed the suppressive effects of *miR-10b* depletion [84]. This indicated that *miR-10b* promotes TGF-β1-induced glioma cell proliferation [84]. Similar to proliferation, the migration and invasion ability of GBM cells was significantly enhanced upon *miR-10b* mimics in combination with TGF-β treatment [84]. Bioinformatics analysis and luciferase reporter assay indicated that *E-cadherin*, *APAF1*, and *PTEN* are the primary targets of *miR-10b* [84]. TGF-β treatment and *miR-10b* mimics individually reduced E-cadherin, APAF1, and *PTEN* protein levels. At the same time, the suppression of *miR-10b* using anti-*miR-10b* enhanced their expression [84]. In vivo, the xenograft tumour model depicted that treatment with TGF-β1 or *miR-10b* agomir significantly promoted GBM tumour growth, whereas the *miR-10b* antagomir remarkably inhibited tumour growth, even in the presence of TGF-β1 [84]. The study demonstrates that TGF-β induces the expression of *miR-10b*, and it promotes TGF-β-mediated proliferation, migration, and invasion in GBM by suppressing *E-cadherin, APAF1*, and *PTEN* [84]. 

#### 2.1.8. *miR-92b*

*miR-92b* levels are elevated, and SMAD3 expression is downregulated in GBM [85]. SMAD3 protein levels were downregulated upon *miR-92b* mimics and were upregulated by *miR-92b* inhibitors [85]. Subsequently, bioinformatics analysis and luciferase reporter assay demonstrated that SMAD3 is a direct target of *miR-92b* [85]. Also, the knockdown of *miR-92b* correlated with the reduced growth of U251 and SHG66 cells and restored G1 accumulation [85]. Knockdown of SMAD3 further decreased the TGF-β-mediated p21 induction [85]. Also, the high expression of *miR-92b* directly downregulates SMAD3 expression and inhibits the TGF-β/SMAD3/p21-mediated reduction in tumour cell growth [85]. Inhibition of *miR-92b* in vivo reduces tumour growth [85]. Hence, in this study, *miR-92b* promotes GBM growth by attenuating the inhibitory effects of TGF-β by targeting SMAD3 and thereby downregulating *p21* [85].

#### 2.1.9. *miR-503*

Analysis of the gene expression omnibus data of glioblastoma samples revealed that *miR-503* is overexpressed in GBM tissue compared to normal tissue [86]. Also, TGF-β treatment significantly increased the expression of *miR-503* in T98G cells through SMAD signalling [86]. *miR-503* overexpression decreased the proliferation, migration, and colony formation ability and restricted apoptosis in GBM [86]. Dual luciferase reporter assay indicated that *PDCD4* is the primary target of *miR-503* [86]. Overexpression of *miR-503* dramatically reduced the mRNA and protein levels of *PDCD4* in GBM cells [86]. Further, the combination of *miR-503* inhibition with varying doses of TMZ showed a synergistic decrease in proliferation and increased apoptosis of A172 and U251 cells, indicated by enhanced cleaved *PARP* [86]. These results suggest that *miR-503* is an oncogenic miRNA induced by TGF-β in GBM [86]. Upon induction by TGF-β, *miR-503* enhances the proliferation, invasion, migration, and drug resistance in GBM by targeting *PDCD4* [86]. 

### 2.2. Tumour Suppressor miRNAs Involved in the TGF-β Pathway in Gliomas

#### 2.2.1. *miR-127-3p*

RNAseq analysis of GBM and normal brain tissues indicated that *miR-127* is down-regulated in GBM tissues compared to normal brain tissues [87]. *miR-127-3p* gene is located in chromosome 14 between two lincRNAs (ENSG00000214548 and ENSG00000258399). DNA methylation and histone acetylase inhibition resulted in the down-regulation of *miR-127-3p* in GBM tissues [87]. Additionally, overexpression of *miR-127-3p* in LN229 and T98G cells reduced the proliferation and caused cell cycle arrest compared to control cells. However, the overexpression of *miR-127-3p* did not significantly affect the apoptosis of glioma cells. In vivo nude mice model with *miR-127-3p* overexpressing LN229 cells displayed reduced colony formation and tumour volume compared to the control group. Hence, *miR-127-3p* functions as a tumour suppressor in GBM [87]. Bioinformatics analysis and luciferase reporter assays revealed that *SKI, RGMA*, *ZWINT*, *SERPINB9*, and *SFRP1* are the primary targets of *miR-127-3p*. SKI is an essential negative regulator of the TGF-β pathway [87]. It binds to SMAD proteins, blocking the ability of the SMAD complexes to activate TGF-β signalling in GBM. Overexpression of *miR-127-3p* or knockdown of *SKI* promotes TGFBR1 expression, phosphorylation of SMAD3, and induces cell cycle arrest of LN229 cells [87]. Hence, *miR-127-3p* is an essential tumour suppressor miRNA that attenuates GBM proliferation by inhibiting the TGF-β signalling [87].

#### 2.2.2. *miR-564*

*miR-564* is downregulated in glioma cells and tumour tissues compared to normal human astrocytes and normal brain tissues, respectively [88]. *miR-564* mimics decreased the proliferation and invasion of U87 cells [88]. Bioinformatics analysis and luciferase reporter assay indicated that TGF-β1 is the primary target of *miR-564* [88]. *miR-564* overexpression markedly decreased the mRNA and protein levels of TGF-β1 [88]. In addition, *miR-564 also* reduced the expression of SMAD4 protein and phosphorylated SMAD2 protein levels [88]. Moreover, protein levels of EGFR and MMP9 were also significantly reduced upon *miR-564* overexpression in U87 cells [88]. EGFR and MMP9 are upregulated in GBM tissues compared to normal brain tissues [88]. Also, a significant negative correlation was observed between *miR-564*, *EGFR*, and *MMP9* [88]. Cell proliferation and invasion assays indicated that the increase in proliferation and invasion by TGF-β treatment was attenuated by *miR-564* overexpression [88]. Further, the U87-engrafted in vivo GBM tumour model indicated a reduction in tumour growth upon *miR-564* overexpression [88]. Also, the mRNA and protein expression of TGF-β1 was lower in *miR-564*- treated tumours than in scramble-treated tumours [88]. Hence, *miR-564* is a tumour suppressor miRNA, which restricts proliferation and invasion in GBM by targeting TGF-β1, and its downstream targets *EGFR* and *MMP9* [88]. 

## 3. LncRNAs Involved in the TGF-β Pathway in GBM 

### 3.1. Oncogenic lncRNAs Involved in the TGF-β Pathway in Gliomas

#### 3.1.1. *LncRNA-ATB*

*LncRNA-ATB* levels are higher in glioma tissues and U251, A172 cell lines than in normal brain tissues [89]. Increased expression of *lncRNA-ATB* correlated with poor survival of GBM patients [89]. Further, loss of function studies depicted a reduced proliferation, migration, and invasion of U251 and A172 cells [89]. The study also indicated a negative correlation between the expression of *lncRNA-ATB* and *miR-200a* in GBM tissues. *miR-200a* is downregulated in GBM tissues and cell lines, and the knockdown of lncRNA-ATB significantly increased *miR-200a* expression in U251 and A172 cells [89]. Luciferase reporter assay and Ago2 pulldown assays validated that *lncRNA-ATB* and TGF-β2 are direct targets of *miR-200a*. Also, *miR-200a* inhibition results in up-regulation of TGF-β2 [89]. *LncRNA-ATB* knockdown mediated a reduction in cell proliferation, colony formation, and invasion of U251 and A172 cells, which is attenuated upon *miR-200a* inhibition. Additionally, the knockdown of *lncRNA-ATB* significantly reduced the levels of TGF-β2 expression, which was rescued by *miR-200a* inhibition. Glioma samples show a positive correlation between *lncRNA-ATB* and TGF-β2 and a negative correlation between *miR-200a* and TGF-β2. The reduction in mRNA and protein levels of TGF-β2 upon *lncRNA-ATB* depletion was further downregulated upon *miR-200a* overexpression. In contrast, TGF-β2 expression was rescued with the combination of *lncRNA-ATB* knockdown and *miR-200a* inhibition [89]. Studies in nude mice models upon *lncRNA-ATB* depletion demonstrated a reduction in tumour volume, tumour weight, and reduced proliferation index indicated by Ki67 staining, supporting the oncogenic role of *lncRNA-ATB* in GBM. These results suggest that *lncRNA-ATB* could competitively bind *miR-200a* to stabilize TGF-β2 and promote TGF-β2-mediated GBM pathogenesis [89]. 

Another study by Tang et al. reported that *lncRNA-ATB* is upregulated by TGF-β1 treatment in LN18 and U251 cells [90]. The up-regulation of *lncRNA-ATB* by TGF-β treatment was abrogated upon treatment with TGF-β inhibitor, SB505124, indicating the SMAD2/3 mediated regulation of *lncRNA-ATB* expression [90]. *LncRNA-ATB* overexpression combined with TGF-β1 treatment increases the invasion of U251 and LN18 cells. Also, *lncRNA-ATB* overexpression and TGF-β1 treatment promote the phosphorylation of *p65*, the nuclear translocation of *p65*, and the phosphorylation of *p38*. These results indicate the activation of the *NF-κB* and *p38/MAPK* pathways by TGF-β regulated *lncRNA-ATB* [90]. The increase in invasion in LN18 and U251 cells upon lncRNA-ATB overexpression and TGF-β treatment was significantly abolished upon treatment with *NF-κB* and *p38/MAPK* pathway inhibitors. These results suggest that the SMAD2/3 transcription factor induces *lncRNA-ATB* expression in GBM, and it promotes TGF-β-mediated GBM invasion through the *NF-κB* and *p38/MAPK* pathways. 

#### 3.1.2. *LncRNA-UCA1*

TGF-β significantly upregulates *lncRNA-UCA1* expression in U87 and U251 cells [91]. Further, the knockdown of *lncRNA-UCA1* attenuated the invasion and stemness of glioma cells induced by TGF-β [91]. Particularly, *lncRNA-UCA1* knockdown downregulates SLUG, ALDH1, and NANOG, which are involved in TGF-β up-regulation. Luciferase reporter assay and Ago2 RIP suggest direct binding of *lncRNA-UCA1*, *miR-1*, and *miR-203a*. Moreover, *miR-1* and *miR-203a* directly mediate SLUG down-regulation. The study also reported a positive correlation between *lncRNA-UCA1* and SLUG expression in GBM tissues. *LncRNA-UCA1* promotes SLUG expression by binding to and titrating *miR-1* and *miR-203a*. Reduction in spheroid formation, SLUG expression, and ALDH1 activity upon *lncRNA-UCA1* knockdown is partially rescued upon Slug overexpression. Hence, these results suggest that TGF-β induced *lncRNA-UCA1* acts as a molecular sponge for *miR-1* and *miR-203a* to promote SLUG expression and SLUG-mediated GBM cell stemness [91]. 

#### 3.1.3. *LINC00645*

TCGA and GSEA data analysis by Li et al. indicated that *LINC00645* is upregulated in GBM patients compared to normal brain tissues [92]. Expression of *LINC00645* was high with increasing grades of glioma. Moreover, TGF-β induces *LINC00645* expression in GBM [92]. Knockdown of *LINC00645* attenuated the malignant behaviour of GBM by decreasing proliferation, invasion, migration, and EMT in T98G and U251 cells. Notably, *LINC00645* knockdown reduces ZEB1 levels, an essential target of *miR-205-3p* [92]. *miR-205-3p* is downregulated in GBM tumour tissues and GBM cell lines compared to normal brain tissues [92]. Low expression of *miR-205-3p* indicates poor survival in GBM patients [92]. TGF-β treatment significantly downregulated *miR-205-3p*, and a negative correlation is observed between *LINC00645* and *miR-205-3p* in GBM patients’ samples [92]. *miR-205-3p* overexpression significantly reduced the levels of *LINC00645*, while depletion of *LINC00645* promoted *miR-205-3p* expression. Luciferase reporter assay and Ago2-RIP indicate a direct interaction between *LINC00645* and *miR-205-3p* [92]. Epithelial marker, E-cadherin expression is downregulated, and expression of mesenchymal markers, vimentin, N-cadherin, SNAIL, and ZEB1 is upregulated upon TGF-β treatment in U251 and T98G cells. Whereas knockdown of *LINC00645* in combination with TGF-β treatment increased E-cadherin levels and reduced the expression of mesenchymal markers. Further, TGF-β treatment in combination with *miR-205-3p* overexpression reversed these effects [92]. *miR-205-3p* is a tumour suppressor that directly targets and degrades ZEB1 [92]. TGF-β induced *LINC00645* mediated GBM invasion, and migration is reversed upon *miR-205-3p* overexpression [92]. These results indicate that *LINC00645* sponges *miR-205-3p* to stabilize ZEB1 and promote GBM pathogenesis [92]. In addition to its effect on GBM invasion, migration, and EMT, *LINC00645* also induces stemness in GBM. Western blotting analysis upon *LINC00645* depletion reduced the expression of stemness factors, BMI-1, OCT-4, SOX-2, and NANOG [92]. Sphere-forming assay indicated a decrease in sphere-forming ability on *LINC00645* knockdown. In contrast, *LINC00645* overexpression had the opposite effect [92]. Also, *LINC00645* depletion partly decreased NESTIN expression and increased the GFAP expression in U251 cells [92]. Depletion of *LINC00645* reduced tumour growth of the tumour xenograft model in vivo [92]. *LINC00645*/*miR-205-3p*/ZEB1 axis regulates invasion, migration, and EMT in GBM, and *LINC00645* also promotes stemness in GSCs [92]. 

#### 3.1.4. *LINC00115*

RNA-sequencing of TGF-β treated GSCs revealed up-regulation of *LINC00115* [93]. Moreover, *LINC00115* expression is higher in GBM tumour samples than in normal tissues and correlates with poor patient prognosis [93]. *LINC00115* knockdown inhibits GSC proliferation and neurosphere formation in vitro and also inhibits tumour formation in the xenograft model [93]. *LINC00115* physically associates with *miR-200b* and *miR-200c* [93]. Also, *LINC00115* depletion reduced the expression of ZEB1 and ZNF596 and reduced invasion in GSCs [93]. Reporter assays indicate that ZEB1 and ZNF596 are targets of *miR-200b* and *miR-200c*. Down-regulation of ZEB1 and GBM invasion upon *LINC00115* knockdown was reversed upon *miR-200b* overexpression, indicating that *LINC00115* competitively binds to *miR-200b* to promote ZEB1-mediated GBM invasion [93]. *LINC00115* and its target *ZNG596* are co-expressed in clinical glioma samples. Concomitantly, exogenous expression of *ZNF596* in *LINC00115*-depleted GSCs reversed the inhibition of cell proliferation caused by *LINC00115* depletion, indicating that ZNF596 is the downstream effector of *LINC00115*-driven GBM tumourigenicity [93]. Also, *LINC00115* binds to *miR-200* to promote the expression of *ZNF596* [93]. CRISPR-mediated knockout of *ZNF596* indicated that *EZH2* is a direct target of *ZNF596*. *ZNF296* is a transcription factor promoting the expression of *EZH2* [93]. *LINC00115* depletion results in loss of *EZH2* expression, which is reversed upon ZNF596 overexpression [93]. *LINC00115* further activates *STAT3* downstream of *EZH2* through *ZNF596*, indicating that *LINC00115* activates *EZH2/STAT3* signalling through *ZNF596*, thereby promoting GSC self-renewal and tumourigenicity [93]. *LINC00115* aids GSC’s self-renewal by acting as a ceRNA for transcription factors ZEB1 and ZNF596 by sponging *miR-200* [93]. It also promotes GSC’s tumourigenicity through the *ZNF596/EZH2/STAT3* signal axis [93].

#### 3.1.5. *H19 and HOXD-AS2*

Nie et al. identified eight differentially regulated lncRNAs upon TGF-β treatment (*H19*, *HOXD-AS2*, *LINC00635*, *LINC00277*, *RP11-196G11.2*, *LINC00152*, *MALAT1*, and *LOC100506207*) in D54, P-GBM2 cells [33]. TGF-β induces LncRNAs *H19* and *HOXD-AS2* through SMAD signalling [33]. Further, RIP analysis of TGF-β treated cells depicted enhanced binding of *H19* and *HOXD-AS2* with K-homology (KH) splicing regulatory protein (*KSRP*) [33]. *KSRP* degrades follistatin-like 1 (*FSTL1*) and promotes the maturation of *miR-198* in the nucleus [33]. TGF-β-induced *H19* and *HOXD-AS2* competitively bind to KSRP and thus prevent its nuclear translocation [33]. *miR-198* is a tumour suppressor miRNA that promotes TMZ sensitivity in GBM by downregulating *MGMT* [33]. Overexpression of *KSRP* reversed the *H19* and *HOXD-AS2*-mediated up-regulation of *MGMT* expression, which could reverse TGF-β-induced TMZ resistance [33]. *H19* and *HOXD-AS2* confer TMZ resistance by regulating *miR-198* biogenesis by competing with *KSRP* [33].

#### 3.1.6. *MIR4435-2 Host Gene (MIR4435-2 HG)*

Xu et al. reported the high expression of *MIR4435-2HG* in GBM tissue samples [98]. Loss-of-function studies of *MIR4435-2HG* in U251 and U87 cells decreased cell proliferation, colony formation, migration, and invasion [98]. *In vivo,* nude mice models also show reduced tumour volume and growth upon *MIR4435-2HG* depletion [98]. These results indicate an oncogenic role of *MIR4435-2HG* in GBM [98]. The starbase tool revealed that *miR-1224-5p* targets *MIR4435-2HG* [98]. Also, it was observed that *miR-1224-5p* is downregulated in LN229, U87-MG, and U251 cells compared to normal human astrocytes (NHA) [98]. Luciferase reporter assay confirmed the direct binding of *MIR4435-2HG* and *miR-1224-5p* [98]. Functional rescue experiments revealed that the increase in cell proliferation and colony formation induced by *MIR4435-2HG* overexpression was abrogated upon *miR-1224-5p* mimics, indicating that the *MIR4435-2HG* -*miR-1224-5p* axis promotes GBM pathogenesis [98]. Further, the starbase tool and luciferase reporter assay demonstrated that *TGFBR2*, a critical oncogene, is a direct target of *miR-1224-5p* [98]. Further, *MIR4435-2HG* overexpression promoted the expression of *TGFBR2* at mRNA and protein levels [98]. Also, *miR-1224-5p* inhibition reduced proliferation and colony formation ability in GBM, which was rescued upon TGFBR2 overexpression [98]. In addition, *TGFBR2* knockdown antagonized *MIR4435-2HG* overexpression-induced proliferation and colony formation in GBM, indicating that *MIR4435-2HG* promotes GBM proliferation by sponging *miR-1224-5p* and stabilizing TGFBR2 [98]. Hence, this study suggests an oncogenic role of lncRNA *MIR4435-2HG* in GBM by targeting the *miR-1224-5p*/*TGFBR2 axis* [98].

#### 3.1.7. *LncRNA RPSAP52*

Wang et al. identified that lncRNA *RPSAP52* is overexpressed in GBM tumour samples [97]. High expression of *RPSAP52* is associated with poor survival in GBM patients [97]. Wang et al. also observed a positive correlation between *RPSAP52* and TGF-β1expression in GBM samples [97]. Further, overexpression of *RPSAP52* increased TGF-β1 protein expression, and knockdown of *RPSAP52* exhibited the opposite effect [97]. Overexpression of *RPSAP52* and TGF-β1 individually increased the percentage of *CD133+* cells. Further, the overexpression of TGF-β1 rescued the reduction in the percentage of *CD133+* cells observed upon *RPSAP52* silencing [97]. 

#### 3.1.8. LncRNA Plasmacytoma Variant Translocation-1 (*PVT1*)

Li et al. reported overexpression of lncRNA *PVT1* and down-regulation of p53 in higher grades of glioma compared to normal brain cells [94]. Also, the expression of *PVT1* increased with increasing grades of glioma [94]. Kaplan-Meier survival analysis revealed that high *PVT1* levels are associated with poor survival in GBM patients. Clinical GBM samples show high expression of *PVT1*, TGF-β, and pSMAD2/3 levels and low p53 levels [94]. Further, the knockdown of p53 decreased *PVT1* levels in U373 cells, while overexpression of p53 showed a reverse effect [94]. RIP assay revealed the direct interaction between *PVT1* and p53 [94]. Bioinformatics analysis using lncATLAS provided evidence of the interaction of p53 with the *PVT1* promoter. Also, the dual luciferase reporter assay indicated that p53 binds to the promoter of *PVT1* to attenuate the expression of *PVT1* [94]. Loss-of-function studies demonstrated that the knockdown of *PVT1* reduced proliferation, viability, migration, and invasion, induced cell cycle arrest at S and G2/M phases, and promoted apoptosis in GBM [94]. However, the knockdown of p53 showed the opposite effects [94]. Expression of mesenchymal markers, N-cadherin, MMP-9, and MMP-2 was reduced, and E-cadherin was upregulated upon *lncRNA PVT1* depletion. At the same time, the knockdown of *p53* had a reverse effect. Furthermore, the study demonstrated that lncRNA *PVT1* overexpression increased the transcription activity of the TGF-β, as depicted in the dual luciferase reporter assay, and increased the pSMAD2/3 levels [94]. Knockdown of lncRNA *PVT1* decreased the transcription activity of TGF-β, while p53 knockdown displayed the opposite effects [94]. However, the combined knockdown of *PVT1* and *p53* counteracted the suppressive effects of *p53* on TGF-β activity, indicating that *p53* attenuated the TGF-β/SMAD pathway in GBM by targeting *PVT1* [94]. In vivo nude mice model demonstrated that the knockdown of *PVT1* and p53 individually suppressed the tumour growth [94]. In contrast, the combined knockdown of *PVT1* and *p53* counteracted the tumour-suppressive effects of *p53* in GBM [94]. The levels of TGF-β and pSMAD2/3 were determined from in vivo nude tumour tissues transfected with shRNA against *lncRNA PVT1* or *p53* [94]. *P53* knockdown increased *PVT1* levels, TGF-β, and pSMAD2/3 levels [94]. At the same time, *PVT1* knockdown displayed the opposite effects [94]. Also, the action of *lncRNA PVT1* depletion on TGF-β activity was counteracted by *p53* knockdown [94]. This study demonstrates that *p53* potentially contributes to downregulating the oncogenic *lncRNA PVT1*, thereby suppressing the activation of TGF-β and TGF-β mediated GBM progression by modulating the *lncRNA PVT1*-*TGF-β* axis [94].

#### 3.1.9. *LncRNA-MUF*

Using a genome-wide microarray screen, we identified that *lncRNA-MUF* is induced upon TGF-β treatment in T98G cells [95]. Also, *lncRNA-MUF* induction upon TGF-β treatment is observed across other GBM cell lines—LN229, U87-MG, and LN18. Moreover, levels of *lncRNA-MUF* are elevated in GBM tumour samples, and its expression is associated with poor survival and prognosis [95]. *LncRNA-MUF* induction by TGF-β is completely abolished upon treatment with TGFBR1 inhibitor SB505124 in glioma cells. In addition, the ChIP qPCR assay demonstrates the enrichment of SMAD2/3 antibody in the promoter of *lncRNA-MUF* upon TGF-β stimulation [95]. Loss-of-function assays using siRNA against *lncRNA-MUF* revealed that *lncRNA-MUF* promotes proliferation, migration, and invasion in GBM [95]. In addition, we show that loss of *lncRNA-MUF* sensitizes glioma cells to TMZ-induced cell death [95]. Knockdown of *lncRNA-MUF* downregulated its *cis* oncogene, *CAPRIN2*, and various *trans* genes from the TGF-β ontology group (*VIMENTIN, CTGF, c-MYC,* and *SNAIL1*) [95]. Western blotting analysis of mesenchymal markers revealed the down-regulation of N-cadherin, *VIMENTIN*, and *SNAIL1* upon *lncRNA-MUF* knockdown in T98G and U87-MG cells [95]. Bioinformatics analysis and dual luciferase reporter assay demonstrated the direct interaction between *lncRNA-MUF* and *miR-34a*, and overexpression of *miR-34a* reduces *lncRNA-MUF* expression. *miR-34a* has a potential tumour-suppressor role in glioma by targeting several oncogenes, particularly *SNAIL1*, and it is downregulated in GBM tissues compared to normal tissues [95]. *SNAIL1* is a crucial transcription factor that promotes tumour cell invasion and EMT and is upregulated in GBM. We observed a positive correlation between *MUF* and *SNAIL1* expression in GBM tumour samples [95]. Down-regulation of Snail and reduction in invasion upon *lncRNA-MUF* knockdown was rescued upon *miR-34a* inhibition in T98G and U87-MG cells. These experiments indicate that TGF-β-induced *lncRNA-MUF* sponges *miR-34a* to promote *SNAIL1*-induced invasion in GBM [95]. Our study suggests that TGF-β induced *lncRNA-MUF* promotes GBM invasion through the miR-34a/Snail axis [95]. 

#### 3.1.10. *LINC01711*

We have shown that TGF-β induces *LINC01711* expression in glioma cells and that the levels of *LINC01711* are elevated in GBM tumour samples, and its expression is associated with poor patients’ survival and prognosis [96]. Like *lncRNA MUF*, *LINC01711* is also induced by the SMAD2/3 transcription factors downstream of TGF-β signalling. Down-regulation of *LINC01711* reduces proliferation, migration, and invasion and induces apoptosis in GBM [96]. *LINC01711* knockdown results in downregulating ZEB1, a crucial transcription factor that promotes tumour cell invasion and EMT [96]. *LINC01711* also interacts with *miR-34a*, and ZEB1 is a target of *miR-34a* [96]. Reduction of ZEB1 expression due to *LINC01711* knockdown was rescued upon *miR-34a* inhibition [96]. Further, the invasion assay revealed that *miR-34a* inhibition could reverse the reduction in invasion caused by *LINC01711* knockdown in T98G and U87-MG cells [96]. We also observed that *ZEB1* overexpression could partially reverse the *LINC01711* knockdown-mediated reduction in invasion in GBM [96]. Further, we tested if *LINC01711* could promote TMZ resistance in GBM. *LINC01711* depletion significantly reduced proliferation and increased caspase 3/7 activity in T98G and LN229 cells, suggesting that *LINC01711* promotes resistance to TMZ in GBM. Given the role of ZEB1 in TMZ resistance and that *LINC01711* depletion results in ZEB1 inhibition, we evaluated if *LINC01711* knockdown-mediated sensitization of GBM cells to TMZ-induced apoptosis is associated with a reduction in ZEB1 levels. ZEB1 protein levels were significantly downregulated during TMZ treatment in *LINC01711*-depleted cells compared to cells treated with TMZ alone [96]. We found that in addition to TMZ-mediated apoptosis, *LINC01711* knockdown could induce cisplatin-mediated apoptosis in GBM. Hence, upon induction by TGF-β, *LINC01711* promotes GBM proliferation, migration, invasion, and drug resistance by modulating the *LINC01711/miR-34a/ZEBl* signalling axis [96].

### 3.2. Tumour Suppressor lncRNAs Involved in the TGF-β Pathway in Gliomas

#### 3.2.1. LncRNA *TCONS_00020456*

Tang et al., using a microarray screen, identified 1759 upregulated and 1932 downregulated lncRNAs in U251 cells [99]. Among these differentially expressed lncRNAs, they characterized the most downregulated lncRNA—TCONS_00020456 role in GBM pathogenesis [99]. The expression of *TCONS_00020456* decreased with increasing glioma grades, and the low expression of *TCONS_00020456* indicated poor survival of GBM patients [99]. Further, the siRNA-mediated knockdown of *TCONS_00020456* significantly promoted the invasion and migration of U251 and U87 cells [99]. While overexpression of *TCONS_00020456* significantly inhibited invasion and migration in GBM [99]. Bioinformatic analysis suggests that various mRNAs with oncogenic function negatively correlated with *TCONS_00020456* expression [99]. Among them, SMAD2 and PKCα were the top hits [99]. Further, western blotting analysis revealed that the knockdown of *TCONS_00020456* increased the expression of SMAD2, PKCα, N-cadherin, vimentin, and down-regulation of E-cadherin. Also, the phosphorylation of JNK and ERK was elevated upon *TCONS_0002045* knockdown [99]. The overexpression of *TCONS_0002045* reversed these effects [99]. These results indicate that *TCONS_0002045* abrogates GBM invasion and migration by targeting SMAD2 and PKCα pathways [99]. In vivo, analysis of *TCONS_0002045* in nude mice model revealed a decrease in tumour size and weight in the *TCONS_0002045* overexpression group compared to the *TCONS_002045* knockdown group [99]. In addition, the immunohistochemical staining of tumour tissues from the nude mice indicated increased expression of SMAD2 and PKCα in the *TCONS_002045* knockdown group compared to the *TCONS_0002045* overexpression group [99]. Computational analysis using the miRDB database revealed several miRNAs targeting *TCONS_0002045*, *SMAD2*, and *PKCα* [99]. Among these miRNAs, *miR-1279* was identified as the common miRNA target between the three. *LncRNA TCONS_0002045* abrogates GBM migration and invasion by targeting SMAD2 and PKCα [99]. However, the exact mechanism of down-regulation of *SMAD2* and *PKCα* by *TCONS_0002045* and the role of *miR-1279* needs further investigation. 

#### 3.2.2. LncRNA *RP11-838N2.4*

*RP11-838N2.4* expression is lower in TMZ-resistant cells (U87TR, U251TR) compared to the parental non-resistant cells (U87, U251) [100]. Moreover, *RP11-838N2.4* down-regulation is associated with poor prognosis, a high risk of GBM relapse, and shorter postoperative survival times [100]. Overexpression of *lncRNA RP11-838N2.4* enhances the cytotoxic effects of TMZ in vitro and in vivo [100]. Consequently, TMZ-resistant U251TR cells with high *lncRNA RP11-838N2.4* displayed low levels of *miR-10a* [100]. The lncRNA acts as an endogenous sponge for EphA8 by competing with miR-10a and increasing the levels of EphA8, which promotes apoptosis in glioma cells [100]. Notably, *lncRNA RP11-838N2.4* overexpression hindered the TGF-β pathway independent of miR-10a by reducing mRNA and protein levels of TGF-β1, TGFBR1, SMAD2, SMAD3, and SMAD4 levels [100]. *LncRNA RP11-838N2.4* hinders GBM proliferation and promotes TMZ sensitivity and TMZ-mediated apoptosis by sponging *miR-10a* and stabilizing EphA8. In addition, it downregulates the TGF-β pathway by reducing the expression of the signalling pathway’s components. However, the exact molecular mechanism of *lncRNA RP11-838N2.4*-mediated down-regulation of the TGF-β pathway needs further investigation.

## 4. CircRNAs Involved in the TGF-β Pathway in GBM

### 4.1. Oncogenic circRNAs Involved in the TGF-β Pathway in Gliomas

#### *CircARID1A* 

Li et al., using microarray analysis, identified differentially expressed circular RNAs in GBM tumour tissues [101]. *CircARID1A* was highly abundant in GBM samples and GBM cell lines and was also present in the blood exosomes of GBM patients [101]. shRNA-mediated knockdown of *circARID1A* attenuated the migration and invasion of GBM cells [101]. Also, *MMP 2*, *MMP 9*, and *MMP 14* were downregulated upon *circARID1A* knockdown, indicating that *circARID1A* promotes GBM cell migration and invasion [101]. Bioinformatic analysis revealed that *cicrARID1A* interacts with *miR-370-3p* [101]. FISH experiments demonstrated the co-localization of *circARID1A* and *miR-370-3p* in the cytoplasm [101]. Dual luciferase reporter assay with the plasmid containing *miR-370-3p* binding sites of *circARID1A*, co-transfected with *miR-370-3p* mimics, significantly reduced the luciferase reporter activity [101]. These results suggest a direct interaction between *cicrARID1A* and *miR-370-3p* [101]. *miR-370-3p* expression is downregulated in GBM tissue compared to normal brain tissues [101]. Bioinformatics analysis and luciferase reporter assay showed that *TGFBR2* is an essential target of *miR-370-3p* [101]. RNA pulldown assay with biotin-labelled *miR-370-3p* in U87 cells showed enrichment of *TGFBR2* and *circARID1A*, indicating the possibility of circARID1A/*miR-370-3p*/TGFBR2 axis in GBM [101]. Further, silencing *miR-370-3p* increased the invasion of GBM cells [101]. Also, the knockdown of *circARID1A* attenuated *TGFBR2* levels [101]. *miR-370-3p* inhibition promoted TGFBR2 protein levels, which was reversed upon combined knockdown of *miR-370-3p* and *circARID1A* [101]. GBM cell invasion and migration increased upon *miR-370-3p* inhibition, which was reversed upon combined knockdown of *miR-370-3p* and *circARID1A* [101]. Further, in vivo, the xenograft tumour model indicated reduced tumour growth and reduced TGFBR2 protein levels upon *circARID1A* knockdown. Thus, *circARID1A* promotes GBM invasion by sponging *miR-370-3p* to stabilize *TGFBR2* [101]. 

Chen et al. performed circular RNA sequencing in LN229 and T98G GBM cells and identified several differentially expressed circular RNAs. KEGG and gene ontology analysis of the top-upregulated circular RNAs revealed that TGF-β is a vital pathway regulated by the top-candidate circular RNAs [102]. Further mechanistic studies are required to functionally characterize these candidate circular RNAs and their role in the TGF-β pathway in GBM [102]. 

### 4.2. Tumour Suppressor circRNAs Involved in the TGF-β Pathway in Gliomas

#### *CircCD44* 

Leucine-rich repeat-containing 4 (*LRRC4*) is a tumour suppressor in GBM [103]. Feng et al. identified that *LRCC4* promoted the generation of a circular RNA, *circCD44*, from the *CD44* mRNA by inhibiting the interaction of *CD44* pre-mRNA and *SAM68* [103]. *CircCD44* expression is downregulated in GBM tissues and cell lines [103]. Also, the overexpression of *circCD44* attenuated the proliferation, colony formation, and invasion of GBM cells [103]. In vivo, the xenograft model also depicted a reduced tumour growth upon re-expression of *circCD44* [103]. Bioinformatics analysis and reporter assays revealed that *SMAD6* is an essential target of *miR-326* and *miR-330-5p* [103]. Further mechanistic studies depicted that *circCD44* sponges *miR-326* and *miR-330-5p* to stabilize *SMAD6* [103]. Thus, the *LRRC4/SAM68/circCD44/miR-326/miR-330-5p/SMAD6* signalling axis is an essential regulator of GBM pathogenesis [103]. 

## 5. Discussion

The TGF-β signalling pathway is an attractive therapeutic target for GBM. However, the development of therapeutics targeting the TGF-β pathway has been hindered mainly by its critical regulatory roles in normal physiology and due to its ability to function as both tumour promoter and inhibitor in a context-dependent manner [104]. Hence, there is a need to specifically target tumour-promoting functions of TGF-β. Aberrant TGF-β signalling in GBM alters regulatory ncRNA expression and vice versa to promote GBM pathogenesis. NcRNAs can modulate the TGF-β pathway in GBM in the following ways (i) they act as downstream effectors of the TGF-β pathway, (ii) they can regulate components of the TGF-β pathway, and (iii) they can form a positive or negative feedback loop with the TGF-β pathway. 

Many ncRNAs are in clinical trials for potential biomarkers and therapeutic targets for cancers and other diseases. LncRNA *MFI2-AS1* is in clinical trials for use as a diagnostic biomarker for kidney cancer [105], and lncRNAs *UCA1* and *WRAP53* are in clinical trials for use as diagnostic biomarkers for hepatocellular carcinoma [106]. Results from safety trials of RNA-targeted therapies using ASO against lncRNAs are also promising [107]. Andes-1537, a short single-stranded phosphorothioate-deoxyoligonucleotide against antisense non-coding mitochondrial RNA (ASncmtRNA), was evaluated in phase I clinical trial by subcutaneous administration in patients with solid tumours. The results of this study displayed low toxicity of the oligonucleotide, with significant anti-tumour activity in pancreatic and cholangiocarcinoma patients [107]. A phase I trial for a liposomal mimic of *miR-34a* (MRX34) was carried out in patients with renal-cell carcinoma, hepatocellular carcinoma, melanoma, lung cancer, and gastrointestinal stromal tumours [108,109]. The MRX34 treatment in patients pretreated with dexamethasone displayed significant dose-dependent modulation of miR-34a target genes and manageable toxicity [109]. However, studies on ncRNAs as biomarkers and therapeutic agents in GBM are still in the pre-clinical testing stage. The major challenge associated with delivering RNA-targeted therapies to the brain is to cross the blood-brain barrier. ASO-mediated therapies against ncRNAs are best delivered when conjugated with nanoparticulate formulations [110]. ASO-loaded glucosylated-polyion complex micelles have shown promise to effectively deliver ASOs across the blood-brain barrier through the intravenous route [111]. 

TGF-β pathway promotes TMZ resistance in GBM [33]. Several TGF-β target genes, such as *CTGF*, *ZEB1*, and *SNAIL1*, are reported to promote TMZ resistance [31,32]. Many lncRNAs and miRNAs regulate TMZ-mediated cell death in GBM. For example, lncRNAs *H19*, *HOXD-AS2*, *lncRNA-MUF*, *LINC01711*, *lncRNA RP11-838N2.4*, *miR-210-3p* contribute to TMZ resistance [33,81,95,96,100]. Since TMZ is the preferred choice for glioma treatment, the potential of ASOs against these ncRNAs for promoting TMZ sensitivity should be tested in pre-clinical and clinical studies. 

Furthermore, the non-canonical TGF-β downstream targets, such as NF-κB and *PI3K/AKT/mTOR* pathways, promote GBM pathogenesis. Specific lncRNAs and miRNAs establish a link between TGF-β and these non-canonical signalling pathways. For example, lncRNA-ATB, induced by TGF-β, promotes GBM invasion through the NF-κB and *P38/MAPK* pathways [90]. Also, TGF-β-induced *miR-182* suppresses *CYLD* and promotes sustained activation of NF-κB in GBM [78]. Similarly, the hyperactive NF-κB signalling in GBM promotes the expression of *miR-148a* oncogene. *miR-148a*, upon induction, promotes the GBM pathogenesis by activating the TGF-β signalling by promoting the expression of pSMAD3 and downregulating the negative regulators (QKI and SKI) of the TGF-β signalling [82]. It needs to be tested if targeting such ncRNAs, which modulate crosstalk between multiple oncogenic pathways, can achieve better therapeutic efficacy for GBMs.

Most studies on the TGF-β modulating lncRNAs and circRNAs in GBM have focused on their ability to function as ceRNAs to sponge miRNAs. However, lncRNAs and circRNAs also function by interacting with proteins [49,59]. Further studies are needed to identify RNA binding proteins interacting with these lncRNAs and circRNAs to promote GBM pathogenesis and fully understand their potential as therapeutic targets. Dysregulation of several ncRNAs, such as: *miR-129-2*, *lncRNAs AF086127*, *AF086217*, *AF086391*, *AF119852*, *AK021535*, *AK022370*, *AL050068*, *BC012548*, and *BC041658* occurs in DIPG [112]. However, studies are required to understand the relationship between ncRNAs and the TGF-β pathway in DIPG.

In summary, tumour-promoting ncRNAs involved in the TGF-β pathway have the potential to serve as attractive biomarkers and therapeutic targets for GBM.

## Figures and Tables

**Table 1:** TGF-β regulated oncogenic and tumour suppressor miRNAs in GBM.

miRNA	Expression (up ↑ or down ↓)	Type of Regulation	Function	Mechanism of Action in GBM	Type of Model	Cell Lines	Biomarkers/ Therapeutic Target	Reference
**Oncogenic miRNAs**								
*miR-182*	↑	Regulator of TGF-β signalling	*miR-182* promotes GBM cell growth, colony formation, invasion, and anchorage-independent growth	*miR-182* promotes GBM pathogenesis by activating the NF-κB signalling by suppressing CYLD, a negative regulator of NF-κB	Human, in vitro, and in vivo	U373MG, LN229	+/+	[78]
*miR-15a*	↑	Regulator of TGF-β signalling	*miR-15a* promotes GBM cell invasion and migration	*miR-15a* promotes GBM cell migration and invasion by inhibiting SMAD7	Human, in vitro, and in vivo	SHG139	−/−	[79]
*miR-193b*	↑	Regulator of TGF-β signalling	*miR-193b* promotes GBM cell proliferation	*miR-193b* promotes GBM cell growth by directly targeting SMAD3 and restricting the tumour suppressive effects of SMAD3 through p21 down-regulation	Human and in vitro	U87, and U251	−/+	[80]
*miR-210-3p*	↑	Regulator of TGF-β signalling	*miR-210-3p* promotes GBM cell proliferation, invasion, and TMZ resistance	*miR-210-3p* promotes hypoxia-mediated induction of TGF-β expression. It is induced in hypoxic conditions, and it promotes the transcriptional activity of NF-κB in GBM	In vitro	U87, A172, and HS683 GBM cells	−/−	[81]
*miR-148a*	↑	Regulator of TGF-β signalling	*miR-148a* promotes migration and invasion of GBM cells	*miR-148a* promotes the expression of pSMAD3 by downregulating the expression of negative regulators of TGF-β signalling-QKI and SKI. It induces DNA binding activity of NF-κB; *miR-148a* establishes an essential link between NF-κB and TGF-β signalling in promoting GBM pathogenesis	Human, in vitro, and in vivo	LN18 and U-138MG cells	+/+	[82]
*miR-10a/10b*	↑	Effector of TGF-β signalling	*miR-10a/10b* promotes GBM cell proliferation, invasion	*miR-10a/10b* promotes GBM pathogenesis by inhibiting PTEN downstream of TGF-β	Human and in vitro	U251 and SHG-44 cells	−/−	[83]
*miR-10b*	↑	Effector of TGF-β signalling	*miR-10b* promotes GBM cell proliferation, migration, and invasion	*miR-10b* promotes TGF-β-mediated GBM cell proliferation, migration, and invasion by suppressing E-cadherin, *APAF1*, and *PTEN*	In vitro and in vivo	U87 and U251 cells	−/+	[84]
*miR-92b*	↑	Regulator of TGF-β signalling	*miR-92b* promotes GBM cell proliferation	*miR-92b* Promotes GBM growth by attenuating the inhibitory effects of TGF-β by targeting SMAD3 and thereby downregulating p21	Human, in vitro, and in vivo	U251 and SHG66 cells	−/+	[85]
*miR-503*	↑	Effector of TGF-β signalling	*miR-503* enhances the proliferation, invasion, migration, and drug resistance in GBM cells	TGF-β induces *miR-503* expression. *miR-503* further enhances the proliferation, invasion, migration, and drug resistance in GBM cells by directly targeting PDCD4	Human, in vitro	U251, A172, LN-229, T98G, U87MG, and U-138MG GBM cells	−/−	[86]
**Tumour suppressor miRNAs**								
*miR-127-3p*	↓	Regulator of TGF-β signalling	*miR-127-3p* suppresses GBM proliferation	*miR-127-3p* suppressed GBM cell growth by inhibiting SKI oncogene and activating the tumour suppression effect of TGF-β signalling	Human, in vitro, and in vivo	T98G and LN229 cells	−/+	[87]
*miR-564*	↓	Regulator of TGF-β signalling	*miR-564* attenuates GBM cell proliferation, invasion, and migration	*miR-564* downregulates TGF-β1 and SMAD4. It reduces phosphorylated SMAD2/3 levels in GBM cells	Human, in vitro, and in vivo	U87-MG	−/+	[88]

**Table 2:** TGF-β regulated oncogenic and tumour suppressor lncRNAs in GBM.

LncRNA	Expression (up ↑ or down ↓)	Type of Regulation	Function	Mechanism of Action in GBM	Type of Model	Cell Lines	Biomarkers/Therapeutic Target	Reference
**Oncogenic lncRNAs**								
*LncRNA-ATB*	↑	Regulator of TGF-β signalling	*LncRNA-ATB* promotes cell proliferation, colony formation, and invasion of GBM cells	*LncRNA-ATB* is induced by TGF-β. It competitively binds *miR-200a* to stabilize TGF-β2 and promotes TGF-β2-mediated GBM cell proliferation and invasion	In vitro and in vivo mouse model	U251, and A172	+/+	[89]
*LncRNA-ATB*	↑	Regulator of TGF-β signalling	*LncRNA-ATB* promotes GBM cell invasion	*LncRNA-ATB* is induced by TGF-β. It promotes TGF-β-mediated GBM cell invasion through the NF-κB and P38/MAPK pathways	Human—in vitro	LN18 and U251	−/−	[90]
*LncRNA-UCA1*	↑	Effector of TGF-β signalling	*LncRNA-UCA1* promotes invasion and stemness of glioma cells	*LncRNA-UCA1* is induced by TGF-β. It Acts as a molecular sponge for *miR-1* and *miR-203a* to promote *Slug* expression and *Slug*-mediated GBM cell stemness	in vitro	U87 and U251	−/−	[91]
*LINC00645*	↑	Effector of TGF-β signalling	*LINC00645* promotes glioma cell proliferation, invasion, migration, and stemness	*LINC00645* is induced by TGF-β and acts as a molecular sponge for *miR-205* to stabilize ZEB1	Human, in vitro, and in vivo	T98G and U251	+/+	[92]
*LINC00115*	↑	Effector of TGF-β signalling	*LINC00115* aids GSC’s self-renewal by acting as a ceRNA for transcription factors ZEB1 and ZNF596 by sponging *miR-200*. It also promotes GSC’s tumourigenicity through *ZNF596/EZH2/* *STAT3* signal axis	*LINC00115* is induced by TGF-β. It Competitively binds to *miR-200 and* promotes stemness in GSCs by stabilizing ZEB1. It also binds to *miR-200* to stabilize ZNF596, and promotes stemness in GSCs through the *ZNF596/EZH2/STAT3* signal axis	Human, in vitro, and in vivo	U87, LN229, LN18, and T98G	−/+	[93]
*H19,* and *HOXD-AS2*	↑	Effector of TGF-β signalling	*H19* and *HOXD-AS2* promote TMZ resistance in GBM cells	*H19* and *HOXD-AS2* are induced by TGF-β. They confer TMZ resistance by regulating the biogenesis of tumour suppressor miRNA, *miR-198*, by competing with *KSRP*	In vitro	D54, P-GBM2 cells	−/+	[33]
*LncRNA-PVT1*	↑	Regulator of TGF-β signalling	*LncRNA-PVT1* promotes GBM cell viability, proliferation, migration, invasion, and restricts apoptosis of GBM cells	*LncRNA-PVT1* promotes phosphorylation of SMAD2/3 and GBM progression	In vitro and in vivo mouse model	HS683, T98G, U373, SHG44, A172, U251, and U87-MG	−/-	[94]
*LncRNA-MUF*	↑	Positive feedback loop with TGF-β signalling	*LncRNA-MUF* promotes GBM cell proliferation, migration, invasion, and TMZ resistance and restricts apoptosis	*LncRNA-MUF* is induced by TGF-β and promotes GBM cell invasion by sponging *miR-34a* and by stabilizing SNAIL1. It also promotes phosphorylation of SMAD2/3	In vitro	T98G, U87-MG, LN229, and LN18	+/+	[95]
*LINC01711*	↑	Regulator of TGF-β signalling	*LINC01711* promotes GBM cell proliferation, migration, invasion, TMZ resistance and restricts apoptosis	*LINC01711* is induced by TGF-β. It promotes GBM cell invasion by sponging *miR-34a* and by stabilizing ZEB1	In vitro	T98G, U87-MG, LN229, and LN18	+/+	[96]
*LncRNA RPSAP52*	↑	Regulator of TGF-β signalling	*LncRNA RPSAP52* promotes stemness in GBM cells	*LncRNA RPSAP52* promotes stemness in GBM by promoting TGF-β1 expression	Human, in vitro	U373	−/−	[97]
*MIR4435-2HG*	↑	Regulator of TGF-β signalling	*MIR4435-2HG* promotes GBM cell proliferation, colony formation, migration, and invasion	*MIR4435-2HG* promotes GBM cell proliferation by sponging *miR-1224-5p* and by stabilizing the expression of TGFBR2	In vitro, and in vivo	U251, and U87-MG	−/+	[98]
**Tumour suppressor lncRNAs**								
*LncRNA—TCONS_00020456*	↓	Regulator of TGF-β signalling	*LncRNA—TCONS_00020456* promotes the expression of mesenchymal markers in GBM cells and promotes invasion and migration of GBM cells	*LncRNA—TCONS_00020456* promotes invasion in GBM cells by phosphorylating SMAD2/3	In vitro	U251	+/−	[99]
*LncRNA RP11-838N2.4*	↓	Regulator of TGF-β signalling	*LncRNA RP11-838N2.4* restricts GBM cell proliferation and mediates TMZ sensitivity	*LncRNA RP11-838N2.4* downregulates expression of TGF-β1, TGFBR1, SMAD2, SMAD3, SMAD4	In vitro and in vivo	TMZ-resistant GBM cells (U87TR, U251TR)	+/−	[100]

**Table 3:** TGF-β regulated oncogenic and tumour suppressor circular RNAs in GBM.

Circular RNA	Expression (up ↑ or down ↓)	Type of Regulation	Function	Mechanism of Action in GBM	Type of Model	Cell Lines	Biomarkers/ Therapeutic Target	Reference
**Oncogenic Circular RNA**								
*CircARID1A*	↑	Regulator of TGF-β signalling	*CircARID1A* promotes GBM cell migration and invasion	*CircARID1A* promotes GBM invasion by sponging tumour suppressor *miR-370-3p* to stabilize *TGFBR2*	Human, in vitro, and in vivo	U87-MG, and U118	+/−	[101]
**Tumour suppressor circular RNA**								
*CircCD44*	↓	Regulator of TGF-β signalling	*CircCD44* attenuates GBM cell proliferation, colony formation, and invasion	CircCD44 sponges *miR-326* and *miR-330-5p* to stabilize SMAD6	Human and in vitro	Primary GBM cell lines 1104, 1124c, and 1216	+/−	[102]
